# Intraoral scanners for capturing the palate and its relation to the dentition

**DOI:** 10.1038/s41598-021-95103-6

**Published:** 2021-07-29

**Authors:** Jonas Winkler, Nikolaos Gkantidis

**Affiliations:** Department of Orthodontics and Dentofacial Orthopedics, University of Bern, Freiburgstrasse 7, CH-3010 Bern, Switzerland

**Keywords:** Oral anatomy, Outcomes research

## Abstract

Proper superimposition of intraoral scan generated 3D models enables detailed assessment of soft and hard tissue surface changes. This requires accurate 3D models and stable structures as superimposition references. In the maxilla, different reference areas have been proposed, mostly located at the palatal region. In this in vivo study we evaluated the precision of two intraoral scanners (TRIOS 3, 3Shape and CS 3600, Carestream) at the maxilla, focusing on the palate itself and also on its spatial relation to the dentition, following palatal superimposition. Precision was tested through the superimposition of repeated scans on the palate and the dental arch. Overall, the median precision of both scanners was high (< 0.1 mm). Scanner precision was comparable when the palatal area was tested individually. However, TRIOS 3 showed higher precision regarding the assessment of the dental arch, following superimposition of repeated models on the palate (median difference: approximately 40 μm). In few cases, local areas of higher imprecision were present for both scanners, exceeding 0.3 mm. Thus, scanner precision seems to be high in small, but slightly reduced considering larger areas, with differences between scanners. However, the effect on individual tooth position relative to the palate was for both scanners limited.

## Introduction

The use of intraoral scanners to obtain an imprint of the oral cavity has widely expanded in recent years, as it provides advantages for data processing, while eliminating the need for physical storage space. Among other possibilities, the superimposition of such digital models taken at different time points consists a valuable tool for 3D quantitative and qualitative assessment of soft and hard tissue changes over time. For example, individual tooth movements or changes following periodontal surgery, as well as tooth wear, can be accurately monitored using 3D model superimposition techniques^1–3^.

So far, several techniques have been proposed to superimpose digital 3D models for the assessment of spatial or morphological changes in teeth or alveolar processes^3–6^. Although there is no consensus on the best superimposition reference area, the vast majority of studies use the soft-tissue area that corresponds to hard palate, including the palatal rugae^3,5,7^. Orthodontic treatment may implicate this process even more^8,9^.

The performance of 3D superimposition methods primarily depends on three factors. First, reliable and morphologically stable areas should be available to achieve optimal matching of two serial models on these and assess changes elsewhere. Secondly, a robust matching technique should be applied on these areas to ensure optimal model registration. Finally, accurate digital 3D models that adequately represent the original anatomy consist a prerequisite for the high performance of these methods^3,10^.

Therefore, various studies have tested the accuracy of intraoral scanners focusing from single tooth structures to various other intraoral areas^11–14^. The accuracy of intraoral scanners was found to be similar or even superior to that of alginate impressions, which are regularly used for diagnostic purposes^13,15–17^. However, the focus of most existing studies lies on dental hard tissues. To our knowledge, there is only one study that examined the performance of the soft tissue imaging in the palatal region^18^, considering also the spatial position of the palate relative to the dental arch. Another previous study tested the accuracy of the palatal region relative to the alveolar processes, but it was in vitro and concerned edentulous patients^19^. Accurate imaging of the palate is considered crucial for valid superimposition outcomes, since it has been shown that relatively small artifacts might have a significant effect on superimposition outcomes in individual cases^10^. The study of Zhongpeng et al.^18^ tested the performance of one intraoral scanner compared to that of a conventional PVS (Polyvinyl-siloxane) impression and did not evaluate the effect of scanner imprecision on the assessment of the position of single teeth. Thus, one aim of the present in vivo study was to assess the precision of two different intraoral scanners in the palatal area solely. Additionally, the study aimed to assess scanner precision on the spatial position of the dentition relative to palatal structures. The first aim relates to the need for morphologically stable superimposition reference areas and the second to the usage of palatal structures for the assessment of changes in tooth position, following palatal superimposition.

## Material and methods

Ethical approval was obtained prior to the study by the Ethical Committee of the Canton of Bern, member of the Swiss Association of Research Ethics Committees (ID 2017–01659). The methods were carried out in accordance with the relevant guidelines and regulations. All participants signed an informed consent prior to their enrollment in the study.

### Sample

The data used in this study derived from an existing sample collected and described previously for the assessment of precision and trueness of the whole dental arch^13^. Additional information about this sample can be found there. The previous study included data from 12 participants, since 1 participant was excluded due to slight double contour in few incisal surfaces. For the purposes of the present study, we decided to include this participant, and thus, data from 13 subjects (9 M, 4F; 27–52 years old) were analyzed.

These were adult volunteers fulfilling the following eligibility criteria.

- No extremes of palatal shape, no visible edema in the attached gingiva and on alveolar and palatal mucosa (visual inspection by two investigators).
- No extreme malocclusion patterns, no crossbite, no large asymmetries (visual inspection by two investigators).
- Before or > 2 years after the end of any previous orthodontic treatment.

In the absence of any existing data for the palatal area, a power calculation for the present outcomes was not possible. Thus, we decided to use all available data from the previous study^13^. For that study, sample size was determined for the precision outcome based on existing tooth structure data^20^. As reported previously^13^, we were interested on an effect size of 20 μm. For a power of 90%, an alpha of 0.05 and an SD of 8 μm the effect size having a sample size of 5 is 19 μm. However, to compensate for any heterogeneity in study design and methods, we decided to increase our sample to 13.

### Scanners

As reported previously^13^, two different intraoral scanners were tested. These were the CS 3600 (Carestream, Atlanta USA, Software CS Imaging Version 7.0.23.0.d2) and the TRIOS 3 (3Shape, Copenhagen, Denmark, Software Version 1.4.7.5).

### Data acquisition

The data acquisition process has been thoroughly described in a previous study^13^ of a different aim. The required information to allow deep comprehension of the present study by the readers is also provided below.

The following data acquisition sequence was applied in all subjects.

1. Intraoral scans with CS 3600 (two times).
2. Intraoral scans with TRIOS 3 (two times).
3. Intraoral scan with CS 3600 (one time).

The third step was performed to control for the potential effect of timing within the sequence in the acquired models.

All scans were obtained by the first author who had more than two years of experience with regular clinical use of intraoral scanners. The same investigator performed all the steps of data generation following relevant training and under close supervision by the senior author, as published previously^13^.

For acquisition of the scans, participants were seated on the dental chair in a horizontal position. Following proper tooth drying, five intraoral scans of the upper jaw were obtained. The scans were generated according to the manufacturers’ guidelines. Scanning of the maxilla started with the second molar in the first quadrant and ended at the second molar in the second quadrant. Scanning of palatal soft tissues started at the palatal side of the central incisors and moved distally back to the level of the distal end of the second molars. Before completing the whole scan, missed areas were rescanned, aiming to an unbroken and smooth digital image^13^.

### Superimposition procedure

#### Superimposition reference areas and regions of interest

3D surface models from all intraoral scanners were exported as STL files using specific software (CS 3600: CS Imaging, Version 7.0.23.0.d2; TRIOS 3: Trios, Version 1.4.7.5). These STL-files were then imported into Viewbox 4 software (Version 4.1.0.1 BETA 64, dHAL Software, Kifissia, Greece) for further processing^13^.

**Figure 1:**
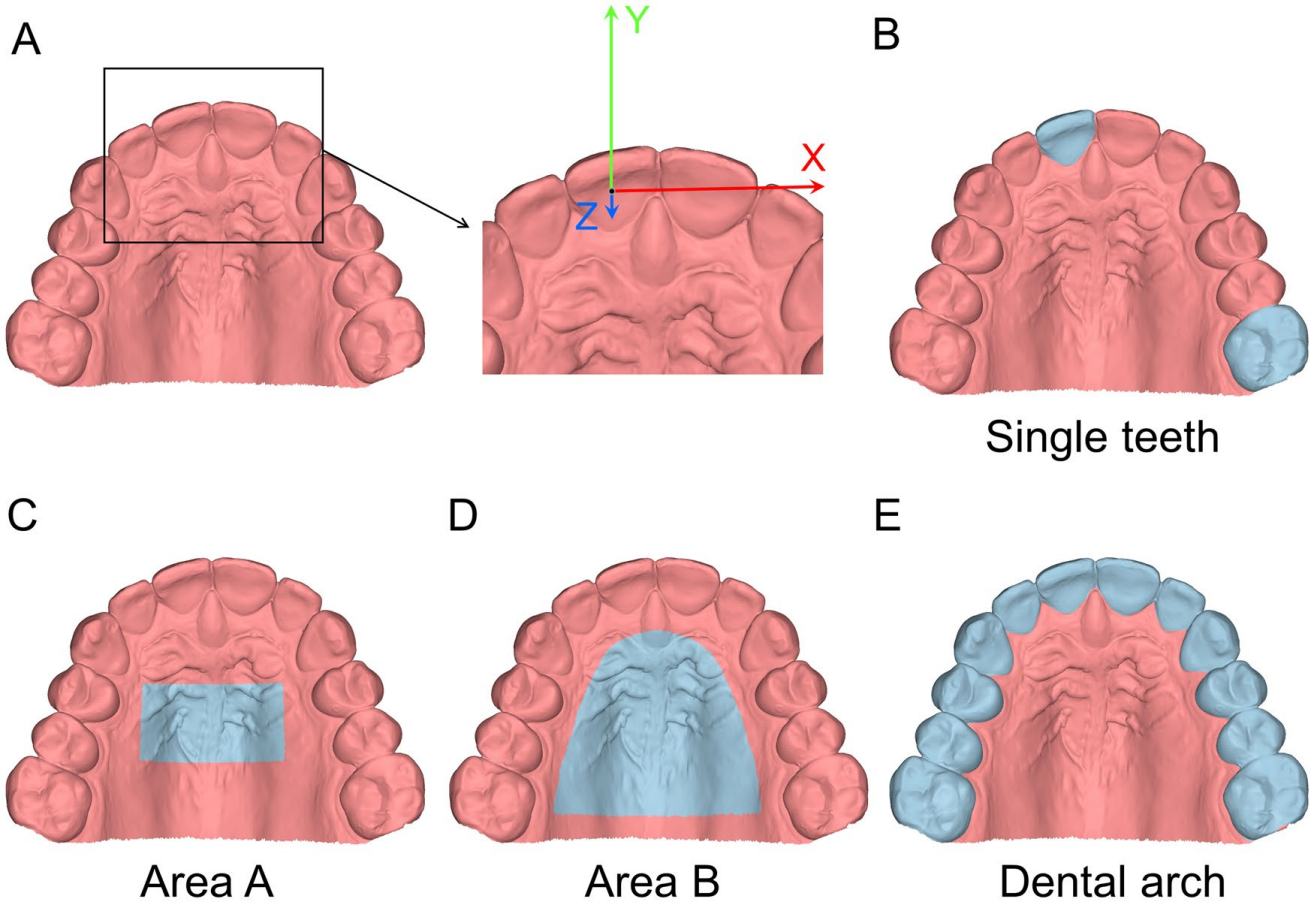
3D model and reference areas used in the study. (**A**) The whole 3D model cropped within 1 mm from the buccal sulcus and distal to the first molar. The black square indicates the area magnified at the right side, showing the axes of movement and rotation, positioned on the crown centroid of upper right central incisor. X-axis: red, lateral movement (positive: left). Y-axis: green, anteroposterior movement (positive: anterior). Z-axis: blue, vertical movement; positive: down). (**B**) Clinical crown of a randomly selected central incisor and the first molar of the contralateral quadrant (light blue). (**C**) Area A including the medial 2/3 of the second and the third rugae and the area 5 mm dorsal to them (light blue). (**D**) Area B including almost the whole palate, delimited by a line 5 mm distant from the gingival margin and extending posteriorly until the middle of the first permanent molars (light blue). (**E**) The dental arch area including all maxillary teeth till the first molars (light blue). All images were generated using Viewbox 4 software (version 4.1.0.1 BETA, http://www.dhal.com/viewboxindex.htm).

The original 3D surface models were manually cropped within 1 mm from the buccal sulcus and distal to the first molar as shown in Fig. 1A. These cropped 3D models, which consisted of approximately 145.000 vertices each, were exported as the final STL files to be analyzed in the study.

For precision testing, three areas were selected separately on the 3D models, by one experienced operator (J.W.), based on strict anatomical definitions and simultaneous visualization of corresponding models. The first (area A) included the medial 2/3 of the second and the third rugae and the area 5 mm dorsal to them. This area is considered relatively stable over time, and therefore, it is widely used as superimposition reference for serial intraoral 3D models to assess morphological changes^3,9^. The second (area B) included almost the whole palate, delimited by a line 5 mm distant from all gingival margins and extending posteriorly until the middle of the 1st permanent molars. This area represents almost the entire palatal structure, which is also used as superimposition reference for serial intraoral models^3,10^. The third area (dental arch) included all maxillary teeth from first molar to first molar to represent the dentition (Fig. 1C–E).

Furthermore, the clinical crowns of a randomly selected central incisor and the first molar of the contralateral quadrant were selected to test the precision of tooth movement assessment, following superimposition on the palate (Fig. 1B).

#### Precision testing

The final 3D models were superimposed in Viewbox 4 using the software’s implementation of the iterative closest point algorithm (ICP)^21^ with the following settings: 100% estimated overlap of meshes, matching point to plane, exact nearest neighbor search, 100% point sampling, exclude overhangs, and 50 iterations. The algorithm was repeatedly applied until the minimum distance between matched models was obtained (usually 4–5 times).

**Figure 2:**
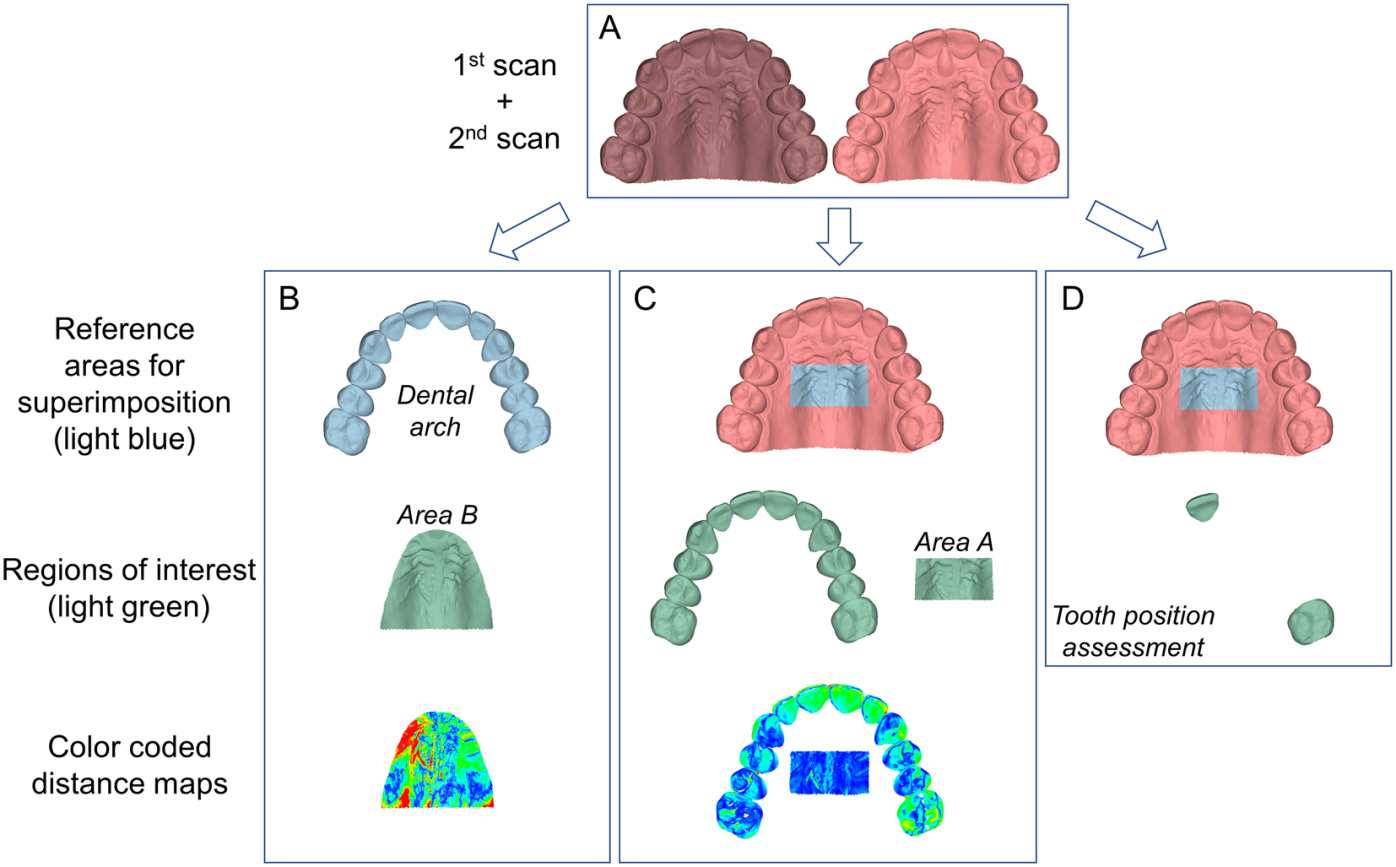
Superimposition and measurement process. (**A**) Repeated scans from the same scanner (either CS3600 or TRIOS3). (**B**) Superimposition procedure of repeated scans on the dental arch and associated assessments. (**C**) Superimposition procedure of repeated scans on area A and associated assessments. (**D**) Superimposition procedure of repeated scans on area A for the evaluation of spatial differences on two corresponding teeth of interest (central incisor and first molar). All images were generated using Viewbox 4 software (version 4.1.0.4 BETA, http://www.dhal.com/viewboxindex.htm).

The precision of intraoral scanners was tested after superimposing the maxillary 3D models obtained from repeated scans of the same scanner (two scans using TRIOS 3 and three using CS 3600), in two different regions. In a first step, the maxillary models were superimposed on the dental arch (reference area). The testing variable was the mean absolute distance (MAD) between the corresponding 3D models at palatal area B (region of interest). In a second step, the maxillary models were superimposed on palatal area A (reference area), always starting from the original 3D model position. In the latter case, the testing variables were the MAD between corresponding dental arches and of palatal area A (regions of interest). In both cases respective color coded distance maps are presented (Fig. 2).

Following each superimposition of repeatedly acquired models in area A, the spatial differences of two corresponding teeth of interest (central incisor and first molar) between the models were assessed separately, as described previously^5^ (Fig. 2). In brief, on the superimposed models, the teeth crowns of interest from the first intraoral scan were superimposed individually, in an additional step, on the respective teeth crowns of the second intraoral scan. Each time, the single tooth movements and rotations associated with the particular tooth superimpositions were measured in three dimensions of space. Through this approach, potential differences in the position of each tooth crown on different scans of the same scanner, were recorded in detail. The origin of the axis of movement was positioned on each crown centroid^22^ and the axes were parallel to the midline palatal suture (Y: green, anteroposterior movement; positive: anterior) and on (X: red, lateral movement; positive: left) or vertical (Z: blue, vertical movement; positive: down) to the occlusal plane. Rotations of each tooth around the X (torque; positive: buccal root), Y (tip; positive: left), and Z (rotation; positive: right palatal) axes were also recorded (Fig. 1A). Zero change in all levels would indicate absolute precision. Any deviation from 0 indicates differences in tooth position at corresponding repeated scans, attributed to scanner imprecision.

### Statistical analysis

Statistical analysis was carried out by using SPSS Software (IBM SPSS Statistics for Windows, Version 25.0. Armonk, NY: IBM Corp.), following a similar approach to a previous analogous study^13^.

Raw data were tested for normality through the Shapiro–Wilk test and did not have a normal distribution in certain cases. Thus, non-parametric statistics were applied^13^.

Differences in the measured variables were tested in a paired manner through the Friedman test. In case of significant outcomes, pairwise comparisons were performed through the Wilcoxon-signed rank test^13^.

In all cases, a two-sided significance test was carried out at an alpha level of 0.05. Bonferroni correction was applied for pairwise a posteriori multiple comparison tests^13^.

## Results

**Figure 3:**
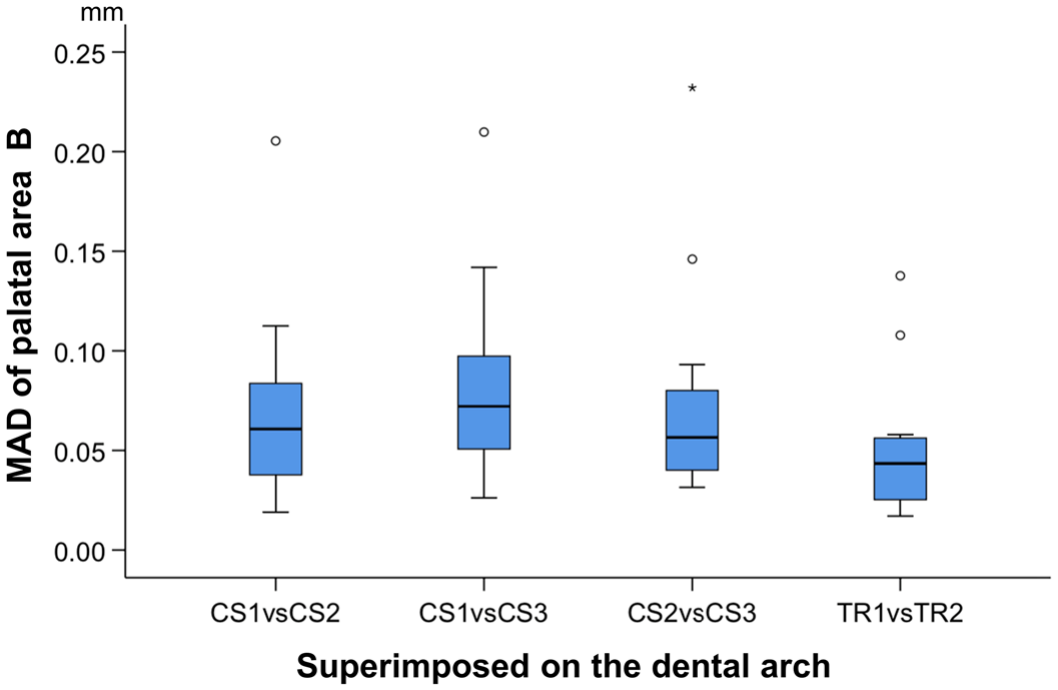
Box plots showing the precision of the two scanners (CS3600 and TRIOS3) measured through the MAD of palatal area B, between repeated scans superimposed on the dental arch (mm; Friedman test, p = 0.176). The upper limit of the black line represents the maximum value, the lower limit the minimum value, the box the interquartile range, and the horizontal line the median value. Outliers are shown as circles or stars, in more extreme cases. CS1, CS2, CS3: CS3600 repeated scans. TR1, TR2: TRIOS3 repeated scans.

**Figure 4:**
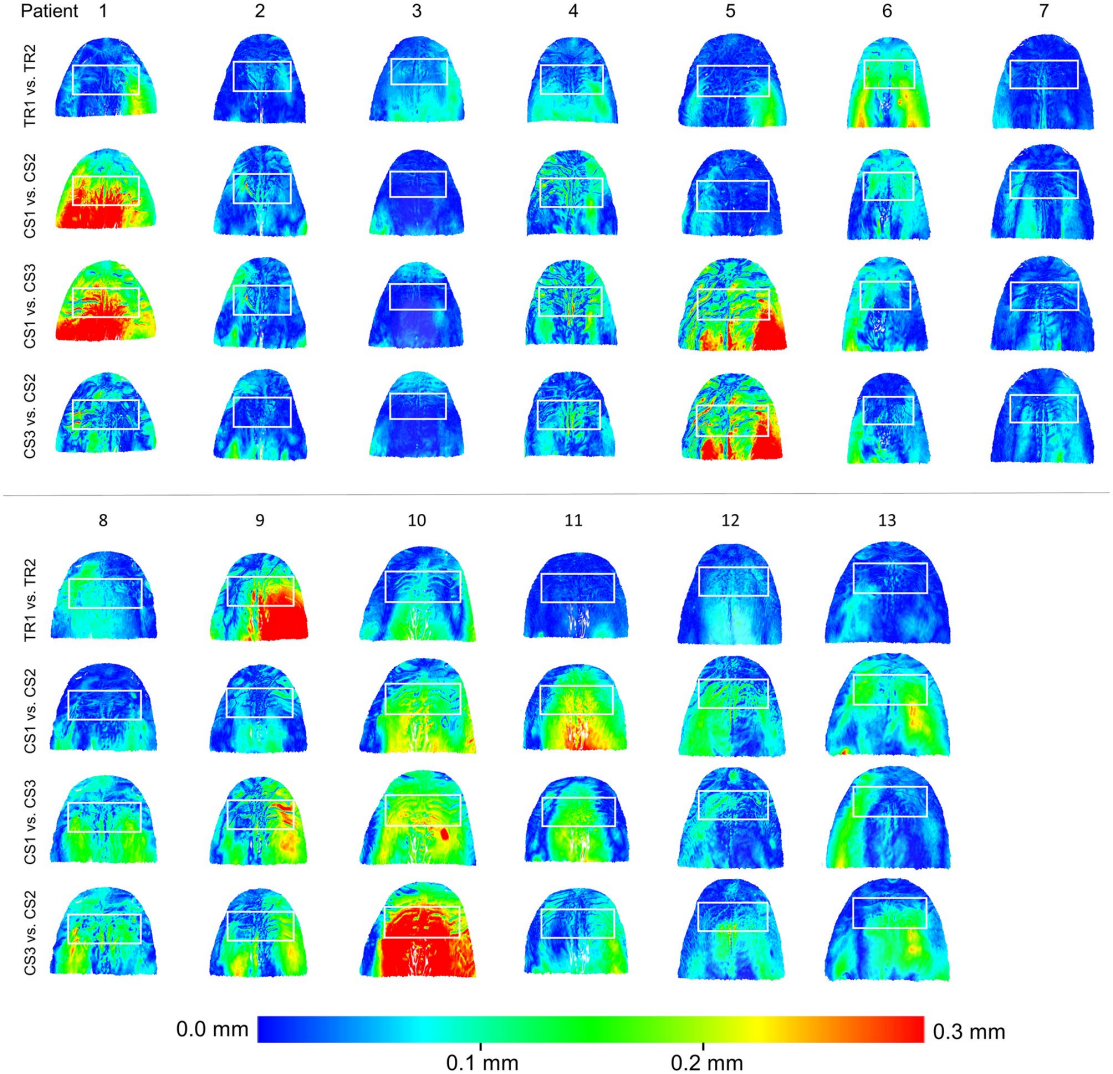
Colour coded distance maps showing the precision of the intraoral scanners in area B, when repeated models were superimposed on the dental arch. Three scans from CS 3600 (CS1, CS2, CS3) and two scans from TRIOS3 (TR1, TR2) were assessed. The white box indicates area A. All images were generated using Viewbox 4 software (version 4.1.0.1 BETA, http://www.dhal.com/viewboxindex.htm).

When superimpositions were performed on the whole dental arch, no significant difference was identified between the precision of the two scanners in palatal area B (Friedman test, p = 0.176; TRIOS 3 median: 0.0434 mm, range: 0.0170, 0.1377 mm; CS 3600 median: 0.0594 mm, range: 0.0191, 0.2321 mm) (Fig. 3). Visual inspection of the respective color maps of both scanners showed no consistent imprecision pattern in terms of amount and location over palatal area B. However, in certain cases the imprecision was more prominent than in others, such as in Patients 1, 5, 9, and 10, depicted in Fig. 4.

**Figure 5:**
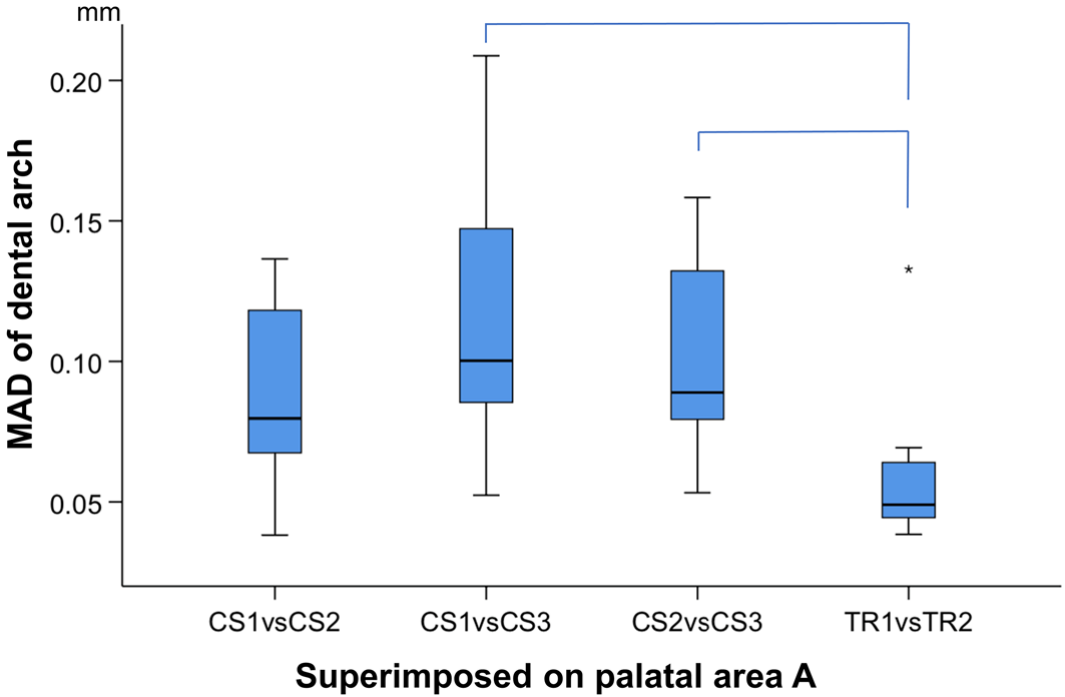
Box plots showing the precision (mm) of the two scanners (CS3600 and TRIOS3) measured through the MAD of the dental arch, between repeated scans superimposed on palatal area A. The upper limit of the black line represents the maximum value, the lower limit the minimum value, the box the interquartile range, and the horizontal line the median value. Outliers are shown as stars, in more extreme cases. Lines connecting pairs of box plots imply significant differences between them (Friedman test, p = 0.000, Wilcoxon Signed Ranks Test, p < 0.008, Bonferroni correction applied). CS1, CS2, CS3: CS3600 repeated scans. TR1, TR2: TRIOS3 repeated scans.

**Figure 6:**
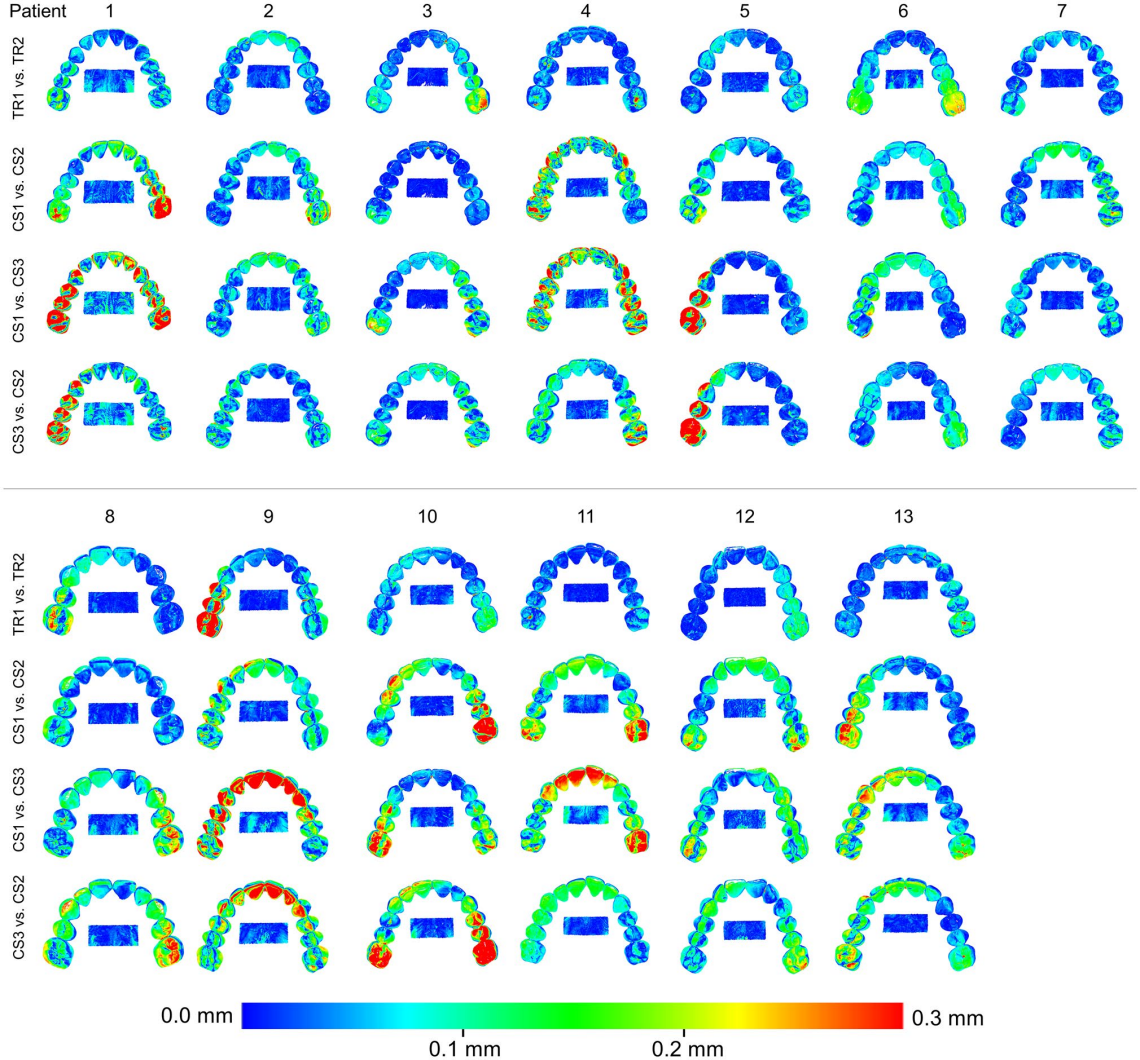
Colour coded distance maps showing the precision of the intraoral scanners on the dental arch area, when superimposed on palatal area A. Three scans from CS 3600 (CS1, CS2, CS3) and two scans from TRIOS3 (TR1, TR2) were assessed. All images were generated using Viewbox 4 software (version 4.1.0.1 BETA, http://www.dhal.com/viewboxindex.htm).

**Figure 7:**
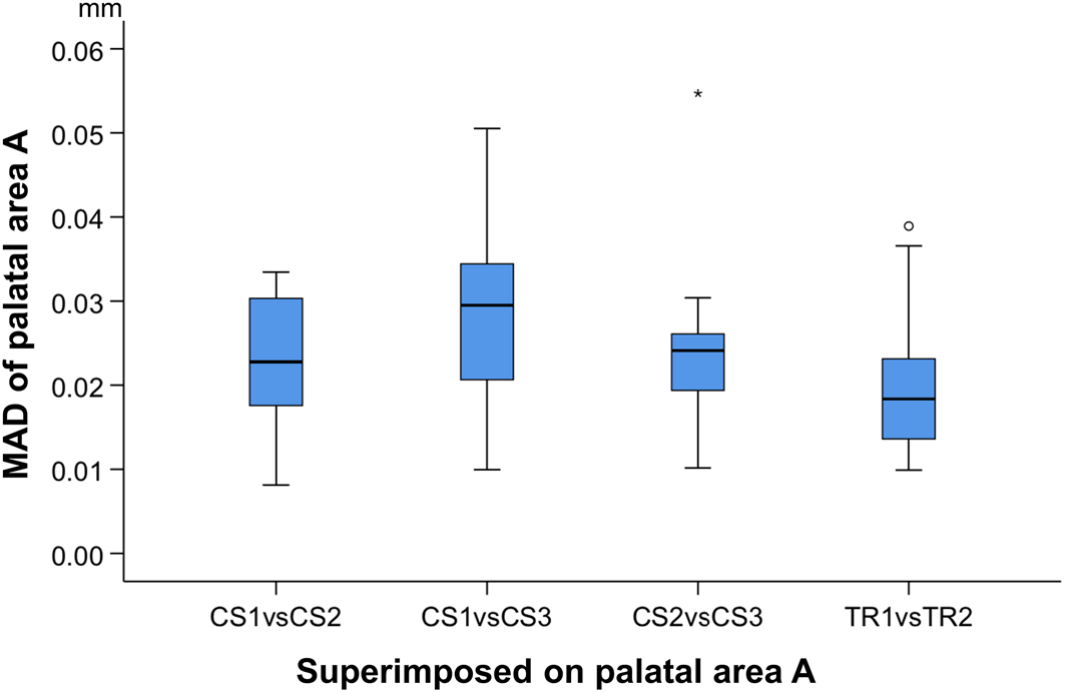
Box plots showing the precision of the two scanners (CS3600 and TRIOS3) measured through the MAD of palatal area A, between repeated scans superimposed on the same area (mm; Friedman test, p = 0.134). The upper limit of the black line represents the maximum value, the lower limit the minimum value, the box the interquartile range, and the horizontal line the median value. Outliers are shown as circles or stars, in more extreme cases. CS1, CS2, CS3: CS3600 repeated scans. TR1, TR2: TRIOS3 repeated scans.

When superimpositions were performed on palatal area A, precision was assessed at: a) the dental arch and b) palatal area A. In the dental arch, significant differences were found between the precision of CS 3600 and TRIOS 3 scanners, with the latter showing better performance (Friedman test, p = 0.000, Wilcoxon Signed Ranks Test, p < 0.008, Bonferroni correction applied) (Fig. 5). The overall difference in the median precision of the two scanners was approximately 40 µm (TRIOS 3 median: 0.0490 mm, range: 0.0384, 0.1328 mm; CS 3600 median: 0.0906 mm, range: 0.0382, 0.2088 mm). When observing the respective colour maps, no specific imprecision pattern was evident. Local imprecision was found to be relatively high, exceeding even 0.3 mm in certain cases, such as in patients 1, 5, 9, and 10, presented in Fig. 6. In few cases, such as in patients 1, 5, and 10, high imprecision seemed to be more often present at the posterior aspects of the dental arch, though this was not consistent (Fig. 6). High precision of a similar level was evident for both scanner types at palatal area A (Friedman test, p = 0.134; TRIOS 3 median: 0.0184 mm, range: 0.0099, 0.0389 mm; CS 3600 median: 0.0244 mm, range: 0.0081, 0.0547 mm) (Fig. 7). Also in this case, the corresponding color coded distance maps show individual variation, with no consistent imprecision pattern within or between subjects. Overall, local imprecisions in area A were relatively small (Fig. 6).

**Figure 8:**
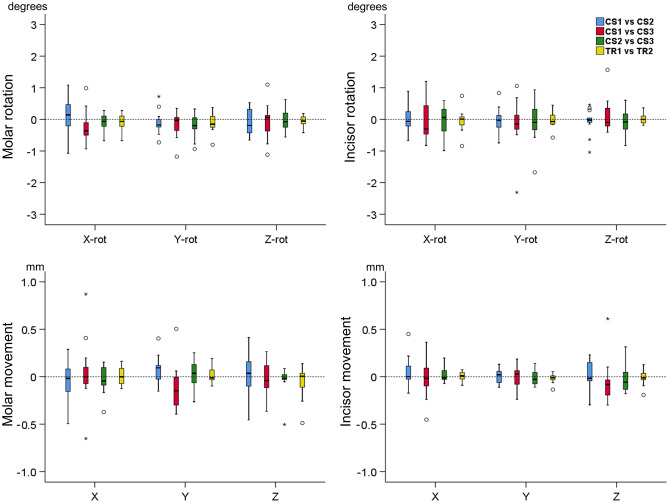
Box plots showing the positional changes of the two teeth of interest (a first molar and a central incisor) in all three planes of space, measured after superimposition of repeated scans on palatal area A (mm for movements, ° for rotations; Friedman test, p > 0.05). The upper limit of the black line represents the maximum value, the lower limit the minimum value, the box the interquartile range, and the horizontal line the median value. Outliers are shown as circles or stars, in more extreme cases. CS1, CS2, CS3: CS3600 repeated scans. TR1, TR2: TRIOS3 repeated scans.

Following superimposition on area A, there were no significant differences in the assessment of tooth position between any pair of repeated scans and for any type of movement, as well as for any tooth tested (Friedman test, p > 0.05). TRIOS 3 scanner showed in most cases less variation from zero, indicating slightly higher consistency in tooth position representation. In the vast majority of cases, imprecision regarding tooth rotations was very low, varying within 1 degree. The imprecision of tooth movement assessment was also very low with most cases showing an imprecision less than 0.25 mm. There was no evidence for systematic error in any of the measured variables (Fig. 8).

## Discussion

The aim of this study was to evaluate the precision of two different intraoral scanners in the palatal area solely and also relevant to tooth structures. Both scanners, TRIOS 3 and CS 3600, showed comparable and very high precision of repeated models considering the palatal rugae area A that is commonly used as superimposition reference^3,5^. However, considering the spatial position of more distant areas within the maxillary model, higher imprecision was observed for both scanners, which was more pronounced for CS 3600. TRIOS 3 showed superior precision in the area of the dental arch, when repeated models were superimposed on the palatal rugae area. This suggests, as shown also previously^13^ for the dental arch, that in smaller areas the two scanners perform similarly, whereas considering larger areas, TRIOS 3 shows slightly better precision. Overall, both intraoral scanners showed good performance, with few 3D models presenting certain sites of higher imprecision, which could exceed 0.3 mm.

Sites of higher imprecision were present, for example, in patients 1, 5 and 10 for CS3600 scanner and in patient 9 for TRIOS3 scanner. As evident in Fig. 4, the higher impression in patient 1 might be attributed to the first CS3600 scan and that in patients 5, and 10 to the third CS3600 scan. Since no specific imprecision pattern was observed between the two scanners, but also neither between nor within patients, the sources of error might be primarily attributed to random factors rather than systematic influences. Such factors can be operator, hardware, or software dependent, with potential interactions among them affecting the outcomes^15,23^.

The effect of the detected amount of imprecision on tooth movement assessment was for both scanners similar and it was considered clinically insignificant. Regarding tooth movement, in all three dimensions of space it was almost always below 0.25 mm. When considering tooth rotations, the amount of error was consistently within 1 degree. No significant differences were observed between tooth types or for the different superimposed scans and there was no evidence of systematic error. However, although differences were not statistically significant, the TRIOS 3 scanner showed in most cases less variation from zero indicating higher consistency in tooth position representation. It should be noted that in certain actual cases, where changes between serial scans are expected, the amount of error detected here might be doubled. This would be the worst case scenario, where at the first scan, one structure is falsely represented towards one direction and at the second scan towards exactly the opposite direction.

We could identify in the literature only one study that tested a hypothesis similar to ours^18^. This study used 3D maxillary models generated after PVS impressions poured with type IV gypsum and compared them to those generated using TRIOS 3 intraoral scanner. Although the study tested the agreement between models generated through different procedures on palatal areas, it did not test the potential effect on the assessment of changes in tooth position. Furthermore, the use of conventional impressions and the procedure of generating stone models might be prone to error, especially considering soft-tissue structures, such as the palate. We suspect that the specific error pattern identified by this study on the palatal area might be due to pressures exerted during impression taking^24,25^. On the contrary, our findings do not suggest any specific pattern of intraoral scanner error on the palatal structures. Furthermore, the congruence of repeated models on area A, when they were superimposed on the same area was very high and comparable to that of the dental arch that was thoroughly tested previously^13^. This does not support the notion of increased errors in the palatal area.

Another previous study tested the overall agreement of an intraoral scanner (TRIOS POD) generated maxillary 3D model with a conventional one^26^. The findings indicated larger deviations between corresponding models in the palatal soft tissue area than in the dentition. However, this finding was not confirmed when precision was tested using repeated intraoral scans. The latter was also evident in our study. The former could be attributed to the process of conventional impression and stone model generation, as discussed above for the study of Zhongpeng et al.^18^. Apart from these considerations, the study of Gan et al.^26^ differs to our study primarily because a whole model superimposition was performed. Thus, the errors that were present in different areas of the model were averaged during the best-fit superimposition process. Furthermore, also that study did not test the effect of scanner error on tooth position assessment.

The literature indicates that most intraoral scanning systems are capable of generating a digital 3D model of acceptable accuracy for diagnosis and several other clinical applications^13–17^. Nevertheless, the assessment of changes in tooth position over time consists probably the most clinically relevant outcome when palatal superimposition of serial dental models is considered^27^. Here, we evaluated differences in the position of certain teeth, following superimposition of repeated scans on an area consisted of the medial 2/3 of the second and the third rugae and the area 5 mm dorsal to them (area A). This area has been widely used as superimposition reference area^3,5^. Furthermore, a recent study suggested that it is suitable to assess anterior tooth movement in orthodontically treated growing patients, without tooth extractions^9^.

The accuracy of intraoral scanners is defined by trueness and precision. Trueness makes a statement about the ability of the intraoral scanner to make an imprint of the oral cavity as close to its true form as possible, without any deformation or distortion. Therefore, trueness requires the presence of the true model, which is quite challenging to be obtained intraorally^13^. As discussed earlier, previous studies reported on trueness based on conventional impressions and stone cast generated models^18,26^. However, this might not be optimal, especially regarding palatal structures, where soft-tissue surface pressure points generated during the conventional impression might alter the original anatomy. Potential effects of this factor have been discussed above. Thus, in our study we only tested precision. Precision indicates the difference between models acquired by repeated scans, under the same conditions (ISO 5725-1). Thus, precision can be tested simply by superimposing repeated scans. We performed in the present study four such comparisons (one for TRIOS 3 and three for CS 3600) allowing a robust estimation of the performance of the intraoral scanners for the tested outcomes. The trueness of these scanners was tested previously for the dental arch, where the true model was possible to be obtained, and proved to be high^13^.

### Limitations

For accuracy testing, a major shortcoming is to generate a highly accurate reference dataset to be used for comparisons. At the moment it is not possible to scan the palate with a high accuracy industrial scanner, due to the size of these scanners relevant to the limited intraoral space. Thus, many studies test solely precision through the reproducibility of direct and indirect digitizations, but fail to assess trueness. That is also the case for the present study. However, the trueness of the specific scanners has been tested previously in the buccal anterior teeth area and proved to be high^13^. Thus, large deviations from the previously defined level of trueness, in other areas of the maxillary model, are not expected, at least for areas of similar extent. Furthermore, the study tested only two commonly used scanners from the wide range of scanners available in the market.

## Conclusions

The present in vivo study demonstrated satisfactory performance of the two intraoral scanners (TRIOS 3 and CS 3600) in terms of precision in the representation of the maxilla, at least for regular clinical use. The precision of both scanners in a small spatial scale was higher compared to that of larger areas. Significant differences between the scanners were found only when larger surfaces were considered, with TRIOS 3 showing slightly higher precision. Overall, no systematic imprecision pattern was evident. In few cases, local imprecisions reached 0.3 mm concerning both scanners. However, the effect of the detected amount of imprecision on single tooth position relative to the palate was for both scanners similar and of limited amount.

## Data Availability

All data are available in the main text or the extended data. The protocols and datasets generated and/or analyzed during the current study are available from the corresponding author on reasonable request.
